# Rapidly progressive cognitive decline and drug-induced encephalopathy secondary to teprotumumab for thyroid eye disease: a case report and narrative literature review

**DOI:** 10.3389/fimmu.2026.1985279

**Published:** 2026-10-22

**Authors:** Zhi-Juan Cheng, Shi-Xian Zhou, Zi-Ying He, Ze-Fang Deng, Chuan Zeng, Wei-Ping Chen, Jiang-Long Tu

**Affiliations:** Department of Neurology, the Second Affiliated Hospital, Jiangxi Medical College, Nanchang University, Nanchang, China

**Keywords:** teprotumumab, thyroid eye disease, drug-induced encephalopathy, IGF-1R, therapeutic plasma exchange

## Abstract

**Background:**

Teprotumumab, a fully human monoclonal antibody targeting insulin-like growth factor 1 receptor (IGF-1R), is an approved targeted therapy for thyroid eye disease (TED). Teprotumumab-associated encephalopathy is an extremely rare but potentially severe central nervous system adverse event, with only three cases reported globally to date. We report the first Han-Chinese patient with Graves’ disease and associated TED complicated by teprotumumab-associated encephalopathy, together with a narrative review of all published cases, to raise clinicians’ awareness and improve early recognition and management of this rare complication.

**Case presentation:**

A 56-year-old Chinese man with Graves’ disease and TED developed acute progressive apathy and encephalopathy characterised by multidomain cognitive and behavioral dysfunction 20 days after the third teprotumumab infusion, with an admission MMSE score of 15. Contrast-enhanced brain MRI, cerebrospinal fluid (CSF) examination, and autoimmune encephalitis antibody screening were unremarkable. Prolonged video-electroencephalography(VEEG) demonstrated intermittent diffuse bilateral slow-wave activity, and widespread interictal epileptiform discharges without electrographic or clinical seizures. Systematic workup excluded infectious, autoimmune, metabolic, and psychiatric encephalopathy. Treatment-emergent hyperglycemia was confirmed as a secondary aggravating factor rather than the primary cause. The patient achieved prominent neurological recovery after individualized treapeutic plasma exchange (TPE) and symptomatic management, with a Naranjo score of 6 indicating probable drug-related encephalopathy.

**Narrative literature review:**

Synthesis of all three previously published teprotumumab-associated encephalopathy cases demonstrates that neurological toxicity can develop as early as the third infusion cycle. These cases present consistent neuroimaging features with variable CSF profiles, and thyroid autoantibody elevation predominantly reflects pre-existing Graves’ disease-related autoimmune background instead of directly mediating neurotoxicity. TPE generally facilitates neurological recovery but may be complicated by procedural hypersensitivity and fluctuating therapeutic responses. Notably, interictal epileptiform discharges on VEEG represent a newly identified neurophysiological manifestation of this rare adverse event. A three-hit interacting pathogenic hypothesis is proposed: impaired IGF-1R-dependent neurotrophic signalling serves as the core injury mechanism, while underlying thyroid autoimmunity and treatment-related metabolic disturbance act as susceptibility-modifying factors.

**Discussion:**

This case expands the ethnic and clinical spectrum of teprotumumab-related encephalopathy. Diffuse interictal epileptiform discharges without ictal changes reflect reactive cortical hyperexcitability secondary to acute encephalopathy. Metabolic disturbance merely exacerbated neurological impairment. Limitations include single-case constraints, simultaneous multi-intervention therapy, and lack of direct intracranial mechanistic evidence.

**Conclusion:**

Teprotumumab-associated encephalopathy is a rare but serious adverse event. Close neurological surveillance and are essential during medication. Accumulated cases are needed to establish unified diagnostic and therapeutic criteria.

## 1 Introduction

Thyroid eye disease (TED), also known as Graves’ ophthalmopathy, is a debilitating autoimmune disorder that can substantially impair patients’ quality of life and remains a major clinical challenge in the management of Graves’ disease (1, 2). Teprotumumab, a fully human monoclonal antibody targeting the insulin-like growth factor 1 receptor (IGF-1R), became the first drug approved by the US Food and Drug Administration (FDA) for the treatment of TED in 2020 (1). Reported adverse reactions to teprotumumab encompass a broad spectrum, including gastrointestinal disturbances such as nausea and diarrhoea, along with muscle spasms, hearing loss, taste disturbance, headache, xerosis, infusion-related hypersensitivity, alopecia, paraesthesia, weight reduction, and hyperglycaemi (1–4). In contrast, teprotumumab-associated encephalopathy is exceedingly rare, with only three cases reported in the literature (4–6). Here, we report a case of teprotumumab-associated encephalopathy presenting with acute neuropsychiatric and cognitive abnormalities in a Han-Chinese patient with TED, with the aim of increasing awareness of this rare but potentially serious neurological adverse event.

## 2 Case presentation

A 56-year-old Chinese man was diagnosed with Graves’ disease in 2024 and with TED on February 25, 2026. The TED diagnosis was based on symptoms of photophobia, lacrimation, and diplopia, together with clinical findings of strabismus and marked bilateral proptosis, and orbital MRI findings of typical extraocular muscle enlargement. The initial Clinical Activity Score was not formally recorded; however, the clinical presentation was consistent with active TED. He received long-term methimazole 5 mg on alternate days for Graves’ disease and fenofibrate for underlying hyperlipidaemia. He was a non-smoker, and had no history of hypertension, diabetes mellitus (baseline HbA1c 5.1%, fasting plasma glucose 5.9 mmol/L), dementia, or other neurodegenerative disorders, and maintained full independence in all activities of daily living before teprotumumab treatment. Teprotumumab infusions were initiated on March 2, 2026 for TED management.

On May 10, 2026, 20 days after his third teprotumumab infusion, his family observed acute onset of rapidly progressive behavioural and neurocognitive abnormalities upon awakening. The presentation was consistent with encephalopathy with multidomain cognitive and behavioural dysfunction. His core symptoms included apathy, hypomimia, bradyphrenia and markedly reduced spontaneous speech. He also developed generalized muscular rigidity, impaired comprehension of verbal commands, predominant recent-memory deficits, and loss of capacity for basic self-care. The patient refused oral food and medications and reported insomnia. Neurological examination revealed he was alert and able to recognise himself and family members but fully disoriented to time, place and recent events. He had blunted affect and soft, monotonous speech. His admission Mini-Mental State Examination (MMSE) score was 15/30, indicating moderate cognitive deficits. Subscores were as follows: orientation 3/10 (marked time-place disorientation), immediate memory 2/3, attention and calculation 3/5, delayed recall 2/3, naming 1/2, repetition 0/1, three-stage command 2/3, reading 0/1, writing 0/1, and figure copying 0/1. Deficits predominated in time-place orientation, with additional involvement of attention, calculation, verbal repetition, reading, writing and visuoconstructive function. Admission Activities of Daily Living (ADL) assessment showed independence only for ambulation and toileting; feeding and dressing required assistance, and bathing needed help. All instrumental activities of daily living, including medication management, financial handling, shopping, public transport and telephone communication, could not be completed independently. The Auditory Verbal Learning Test (AVLT-N1-N3) learning trials yielded recall of zero words on the first and second attempts and two words on the third trial, indicating severe verbal memory-encoding dysfunction during the acute phase of this encephalopathic syndrome. Only the learning component of the AVLT was administered; delayed-recall and recognition subtests were not performed.

Initial laboratory testing showed serum glucose elevated to 14.13 mmol/L with HbA1c of 7.7%, consistent with new-onset hyperglycaemia secondary to teprotumumab exposure. Serum sodium was 138.31 mmol/L and urea 4.12 mmol/L. The calculated plasma osmolality was 294.87 mOsm/kg (reference range, 275–295 mOsm/kg), which did not meet diagnostic criteria for hyperosmolar encephalopathy. Urine ketones were negative. Serum tumour markers, including alpha-fetoprotein, carbohydrate antigen 199 (CA-199), carcinoembryonic antigen (CEA) and total prostate-specific antigen (tPSA), were all within reference ranges. During teprotumumab therapy, high-sensitivity thyroid-stimulating hormone (hs-TSH) decreased markedly from 17.87 mIU/mL to 6.48 mIU/mL, while free triiodothyronine (FT3) and free thyroxine (FT4) remained within normal limits throughout treatment (**Table 1**). Thyroid autoantibody levels were persistently elevated both before and after teprotumumab administration (**Table 1**). Long-term video-electroencephalography (VEEG) showed a poorly regulated posterior dominant rhythm at 8–9 Hz with poor modulation(**Figure 1A**), mild interhemispheric asymmetry, and intact reactivity to eye opening/closure. Intermittent diffuse slow-wave activity was predominant across both hemispheres, with abundant low-to-medium amplitude theta (4–7 Hz) and delta (2–3 Hz) activity (**Figure 1B**, red arrows). Diffuse interictal epileptiform discharges (spikes) were present bilaterally (**Figure 1C**, red arrows), without sustained rhythmic or evolving ictal patterns. No electrographic or clinical seizures were captured, and no myoclonus was observed. Artifacts were carefully excluded. Representative artifact-free segments are shown in **Figure 1**. Brain MRI performed on admission excluded intracranial mass lesions, haemorrhage and cerebral infarction. Teprotumumab was permanently discontinued upon admission. Concomitant medical therapy was initiated, including insulin glargine 10 IU subcutaneously once nightly, short-acting human insulin 6 IU subcutaneously 15 minutes before each meal, methimazole 5 mg orally every other day, fenofibrate 0.1 g orally once daily, and levetiracetam 0.5 g orally twice daily.

**Table 1:** Thyroid autoantibody levels and thyroid function test results.

Test	Reference range	Before teprotumumab therapy	After teprotumumab therapy	Two-month outpatient follow-up
TPOAb, U/mL	<60	>1300	>1300	>1300
TSI, IU/L	<0.55	Not performed	Not performed	1.78
TgAb, IU/mL	<4.0	Not performed	Not performed	Not performed
TRAb, IU/L	0–1.5	22	3.93	1.85
TSH, mIU/L	0.55–4.78	17.87	6.48	5.715
FT4, ng/dL	0.89–1.80	1.42	1.33	1.37
FT3, pg/mL	2.4–4.2	4.06	2.68	2.92

FT3, free triiodothyronine; FT4, free thyroxine; TgAb, thyroglobulin antibody; TPOAb, thyroid peroxidase antibody; TRAb, thyrotropin receptor antibody; TSH, thyroid-stimulating hormone; TSI, thyroid-stimulating immunoglobulin. Not performed indicates that the test was not conducted at the corresponding time point.

**Figure 1:**
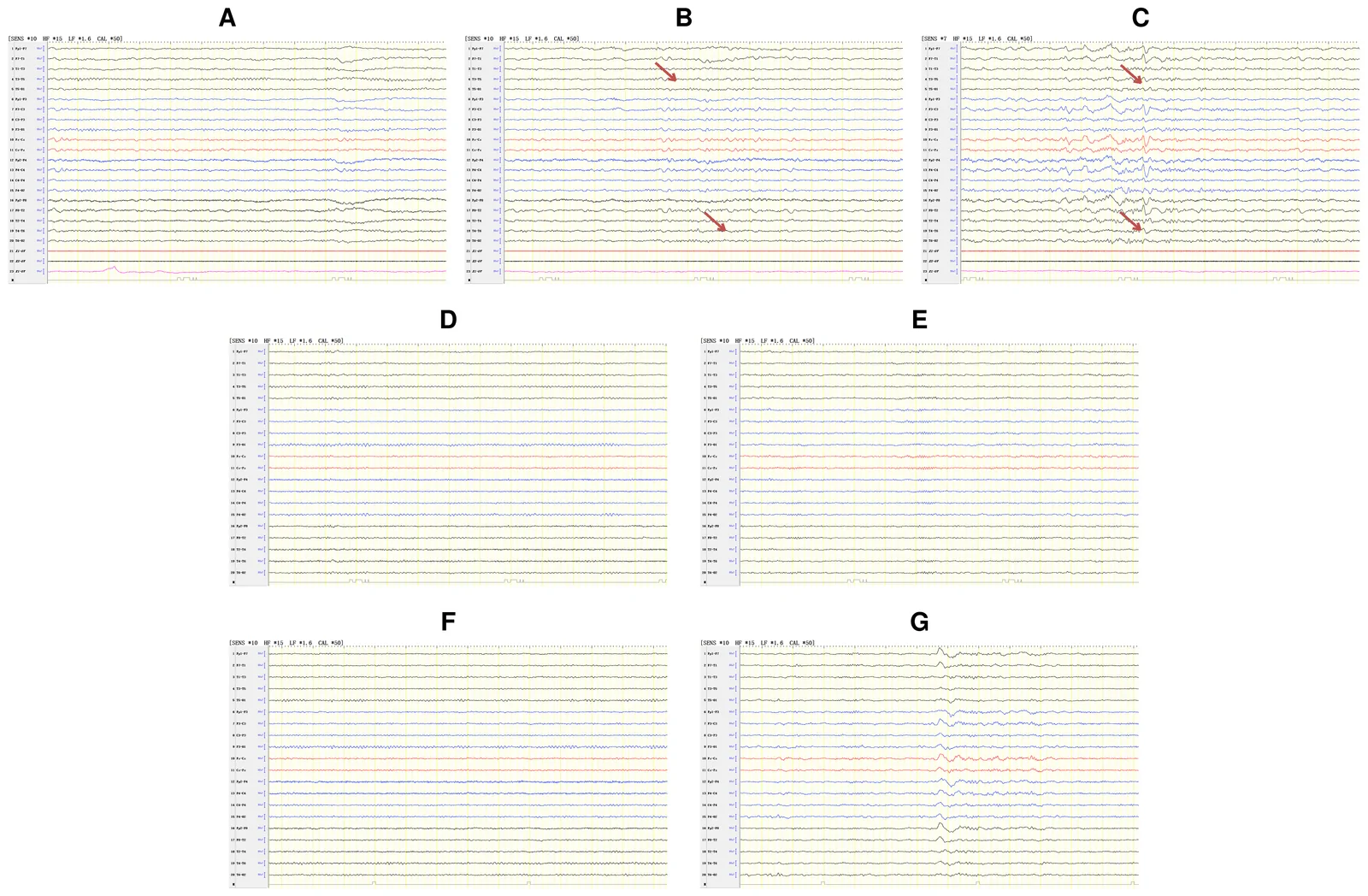
Representative segments of long-term video-electroencephalography (VEEG) (longitudinal bipolar montage; sensitivity, 10 μV/mm; time base, 30 mm/s; low-frequency filter, 1.6 Hz; high-frequency filter, 15 Hz; notch filter, 50 Hz). **(A–C)** on presentation: **(A)** A poorly regulated posterior dominant alpha rhythm (8–9 Hz) with poor modulation and mild interhemispheric asymmetry. Alpha reactivity to eye opening/closing was intact. **(B)** Intermittent diffuse slow-wave activity was predominant across both hemispheres, with abundant low-to-medium amplitude theta (4–7 Hz) and delta (2–3 Hz) activity. Hyperventilation produced further augmentation of diffuse slow-wave activity(slow waves indicated by arrows). **(C)** Diffuse interictal epileptiform discharges (spikes) were present bilaterally, without sustained rhythmic or evolving ictal patterns(spikes indicated by arrows). No electrographic seizures or clinical seizures were captured.Sleep was associated with suppression of the posterior alpha rhythm. **(D, E)** On hospital day 8, after therapeutic plasma exchange and individualised treatment: **(D)** improved posterior dominant alpha rhythm with better organisation and modulation; **(E)** complete resolution of the previously observed diffuse slow-wave activity, with a normalised background rhythm. No interictal epileptiform discharges or electrographic seizures were captured in recording. Representative artifact-free segments are shown. **(F, G)** At 2-month follow-up: sustained normalised background rhythm without recurrence of diffuse slow-wave activity. No interictal epileptiform discharges or electrographic seizures were captured in any recording. Representative artifact-free segments are shown.

On hospital day 1, despite gradual improvement in blood glucose levels under insulin treatment, the patient’s behavioural abnormalities and multidomain neurocognitive deficits remained unchanged, with no symptomatic relief of the underlying encephalopathic syndrome. Contrast-enhanced brain MRI demonstrated no abnormal meningeal or parenchymal enhancement. Lumbar puncture was performed.

Autoimmune encephalitis antibody panels were negative in both serum and cerebrospinal fluid (CSF), and CSF metagenomic next-generation sequencing detected no pathogenic microorganisms (**Table 2**). Additional serological work-up, including ANA, ANA3/ENA profile, ANCA, antiphospholipid panel, rheumatoid factor, vitamin B12 and folate, was unremarkable (**Table 2**). These findings excluded infectious, paraneoplastic, systemic autoimmune, rheumatic, nutritional, electrolyte, renal and hyperosmolar aetiologies of the encephalopathic syndrome.

**Table 2:** Summary of cerebrospinal fluid analysis and systemic laboratory examinations for encephalopathy differential diagnosis.

Category	Test	Method	CSF result	Serum result	Reference range
CSF analysis	Opening pressure	Manometry	85 mmH_2_O	—	70–180 mmH_2_O
	White blood cell count	Automated cell count	0/μL	—	0–5/μL
	Protein	Biochemical assay	0.246 g/L	—	0.15–0.45 g/L
	Glucose	Biochemical assay	6.06 mmol/L	—	2.5–4.5 mmol/L
Autoimmune encephalitis antibody panel	NMDAR, AMPA1/2, LGI1, CASPR2, GABABR1, IgLON5, DPPX, GlyR1, DRD2, mGluR1/5, GAD65, GABA_A receptor α1/β3, neurexin-3α, KLHL11, AQP4, MOG, and GFAP (20 antibodies)	CBA (live-cell CBA for MOG)	Negative	Negative	Negative
Infection screening	Metagenomic next-generation sequencing (mNGS)	NGS	Negative	—	Negative
	Bacterial culture	Culture	Negative	Negative	Negative
	Viral PCR (HSV-1/2, VZV, CMV, EBV, HHV-6, and enterovirus)	PCR	Negative	—	Negative
	Syphilis serology	Chemiluminescence	—	Negative	Negative
	HIV serology	Chemiluminescence	—	Negative	Negative
	Blood culture	Culture	—	Not performed	Negative
	Urine culture	Culture	—	Not performed	Negative
Rheumatological and immunological screening	Antinuclear antibody (ANA)	Indirect immunofluorescence	—	Normal	Negative
	ANA profile 3 (ENA/ANA3)	Immunoblot	—	Normal	Negative
	Anti-neutrophil cytoplasmic antibody (ANCA)	IIF/ELISA	—	Normal	Negative
	Antiphospholipid antibody panel (6 items)	ELISA/CLIA	—	Normal	Negative
	Lupus anticoagulant (LA)	Coagulation assay	—	Normal	Negative
	Rheumatoid factor (RF)	Nephelometry	—	Normal	Negative
Tumour markers	Alpha-fetoprotein (AFP)	CLIA	—	3.5 ng/mL	0–8.1 ng/mL
	Carbohydrate antigen 19-9 (CA 19-9)	CLIA	—	34.90 U/mL	0–37 U/mL
	Carcinoembryonic antigen (CEA)	CLIA	—	3.14 ng/mL	0–5 ng/mL
	Total prostate-specific antigen (tPSA)	CLIA	—	0.16 ng/mL	0–4 ng/mL
Nutritional and metabolic screening	Vitamin B12	CLIA	—	396.0 pg/mL	200–1000 pg/mL
	Folate	CLIA	—	12.1 ng/mL	5.21–20 ng/mL
	Blood ammonia	HPLC/enzymatic	—	19.0 μmol/L	0–71.4 μmol/L
	Serum sodium (Na^+^)	ISE	—	138.31 mmol/L	137–147 mmol/L
	Serum potassium (K^+^)	ISE	—	4.01 mmol/L	3.5–5.3 mmol/L
	Serum calcium (Ca²^+^)	ISE	—	2.20 mmol/L	2.11–2.52 mmol/L
	Blood glucose	Glucose oxidase	—	14.13 mmol/L ↑	3.9–6.1 mmol/L (fasting)
	Urea	Urease assay	—	4.12 mmol/L	2.9–8.2 mmol/L
	Creatinine	Biochemistry	—	89.6 μmol/L	57–97 μmol/L
	Liver function tests (ALT, AST, ALP, GGT, and bilirubin)	Biochemistry	—	Not performed	Normal
	Urine ketones	Dry chemistry	—	Negative	Negative
	Calculated plasma osmolality	2 × Na^+^ + glucose + urea	—	294.87 mOsm/kg	275–295 mOsm/kg

ALP, alkaline phosphatase; ALT, alanine aminotransferase; ANA, antinuclear antibody; ANCA, anti-neutrophil cytoplasmic antibody; AQP4, aquaporin-4; AST, aspartate aminotransferase; CBA, cell-based assay; CEA, carcinoembryonic antigen; CLIA, chemiluminescence immunoassay; CMV, cytomegalovirus; CSF, cerebrospinal fluid; EBV, Epstein-Barr virus; ELISA, enzyme-linked immunosorbent assay; ENA, extractable nuclear antigen; GGT, gamma-glutamyl transferase; GFAP, glial fibrillary acidic protein; HHV-6, human herpesvirus 6; HIV, human immunodeficiency virus; HPLC, high-performance liquid chromatography; HSV, herpes simplex virus; IIF, indirect immunofluorescence; ISE, ion-selective electrode; LA, lupus anticoagulant; mNGS, metagenomic next-generation sequencing; MOG, myelin oligodendrocyte glycoprotein; NGS, next-generation sequencing; PCR, polymerase chain reaction; RF, rheumatoid factor; tPSA, total prostate-specific antigen; VZV, varicella-zoster virus. Not performed indicates that the test was not conducted. An em dash indicates that the test was not applicable to the corresponding specimen.

Based on the clinical phenotype and treatment outcomes from three previously published case reports of teprotumumab-induced encephalopathy (5–7), teprotumumab-mediated encephalopathy with multidomain cognitive and behavioural dysfunction was considered the most plausible diagnosis. Notably, the index 2021 case failed to respond to high-dose intravenous glucocorticoid therapy, with marked resolution of encephalopathic manifestations only achieved after switching to plasma exchange (5). Guided by this positive therapeutic response, the subsequent two reported cases adopted an alternate-day TPE regimen; however, one patient experienced transient clinical fluctuation after the first TPE session (6). To eliminate such disease instability and enable continuous, stable clearance of circulating pathogenic immune mediators, we individualized the therapeutic strategy for the current patient. Accordingly, high-dose steroid pulse therapy was withheld, and a standardized once-daily TPE protocol was initiated on hospital day 2. Substantial clinical improvement occurred within 24 hours after the first TPE session, characterised by resolution of generalized muscular rigidity, normalisation of sleep, increased spontaneous speech, and recovery of independent feeding and dressing. The patient could follow simple commands, although time-place disorientation persisted, and blood glucose improved concurrently.

On hospital day 3, the second TPE cycle was completed without any adverse reactions, accompanied by obvious improvement in time and place orientation. Repeat brain MRI remained unremarkable.

On hospital day 4, the third TPE session was initiated but prematurely terminated due to an acute infusion-related anaphylactoid-like reaction manifested by fever, chills and hypotension; urticaria, angioedema and respiratory compromise were absent. Intravenous fluid resuscitation plus low-dose corticosteroids were administered, which resolved haemodynamic instability; no further TPE cycles were performed. Importantly, despite completion of only two full TPE cycles before the third session was terminated by an adverse reaction, the patient achieved sustained, fluctuation-free improvement of this encephalopathic syndrome. Multidomain neurocognitive function continued to recover steadily under ongoing insulin and levetiracetam therapy, and blood glucose remained within normal limits. Repeat VEEG demonstrated complete resolution of the previously observed diffuse slow-wave activity, with a normalised background rhythm. No interictal epileptiform discharges or electrographic seizures were captured (**Figures 1D–F**). Pre-discharge brain MRI showed no new structural, ischaemic or intracranial lesions.

On hospital day 9, levetiracetam was discontinued, and the patient was discharged. At discharge, the MMSE score improved to 25/30: orientation 8/10 (complete recovery of time orientation with mild residual deficits in fine-grained place information), immediate memory 3/3, attention-calculation 3/5, delayed recall 3/3, naming 2/2, repetition 1/1, three-stage command 2/3, reading 1/1, writing 1/1, and figure copying 1/1. Time orientation, verbal memory, naming, repetition, visuoconstruction, reading and writing nearly returned to baseline. Mild deficits remained in serial subtraction and multi-step executive function. Basic ADL were fully recovered, including ambulation, feeding, dressing, hygiene, bathing, toileting and medication management. Instrumental ADL, such as public transport, cooking, housekeeping, laundry and shopping, remained impaired. Repeat AVLT-N1-N3 showed improved verbal memory encoding, with recall scores of 2, 3 and 4 words across learning trials (total = 9), compared with baseline scores of 0, 0 and 2 words.

At the 2-month follow-up, the therapeutic benefit of teprotumumab for TED remained stable, with sustained neurological recovery without residual sequelae and subjective improvement in proptosis, diplopia, photophobia, and lacrimation. Repeat VEEG demonstrated a sustained normalised background rhythm without recurrence of diffuse slow-wave activity. No interictal epileptiform discharges or electrographic seizures were captured. Representative artifact-free segments are shown in **Figures 1F, G**. Serial thyroid function tests showed a further reduction of TSH to 5.715 mIU/mL. FT3 and FT4 remained within reference ranges, while thyroid autoantibody levels remained substantially elevated.

A detailed timeline of clinical manifestations, interventions, functional recovery, and laboratory findings is provided in **Supplementary Table 3**.

## 3 Discussion

Teprotumumab is a fully humanized monoclonal antibody targeting the insulin-like growth factor-1 receptor (IGF-1R) and serves as a targeted therapy for TED (4). By inhibiting IGF-1R signaling and its functional interaction with the thyroid-stimulating hormone receptor (TSHR) in orbital fibroblasts, teprotumumab attenuates autoimmune inflammatory activity within the orbit and improves characteristic manifestations of TED, including proptosis, eyelid swelling, and diplopia (3, 7). Clinical trials and post-marketing studies have predominantly reported adverse events such as hyperglycemia, muscle spasms, hearing impairment, and gastrointestinal symptoms, whereas severe central nervous system adverse events, including encephalopathy, appear to be exceptionally uncommon (1, 4–7). Consequently, the clinical phenotype, underlying mechanisms, and optimal monitoring and management strategies for teprotumumab-associated encephalopathy remain poorly characterised. Three previously published cases of teprotumumab-associated encephalopathy have been previously reported by Hoang et al. (2021), Yee et al. (2023), and Spicer et al. (2026) (5–7). Herein, we performed a narrative literature review of these three cases and present the fourth worldwide case of teprotumumab-related encephalopathy, which is, to our knowledge, the first documented in a Han-Chinese patient. This case expands the recognized clinical spectrum of this rare neurological adverse event and provides additional clinical evidence to facilitate the recognition, differential diagnosis, and individualized risk stratification of neuropsychiatric complications arising during teprotumumab therapy.

The IGF-1/IGF-1R signalling pathway is widely distributed throughout the central nervous system, including brain regions involved in memory and executive function as well as resident immune cells such as microglia (8, 9). IGF-1 signaling contributes to neuronal survival and plasticity, modulation of neuroinflammation, and the maintenance of learning, memory, and higher-order cognitive function (8). Although IGF-1R is involved in the transport and biological actions of circulating IGF-1 within the central nervous system, the extent to which teprotumumab itself enters the brain and directly inhibits central IGF-1R signaling remains uncertain (5). Nevertheless, previous studies suggest that impaired central IGF-1/IGF-1R signalling constitutes a biologically plausible hypothesis for teprotumumab-associated encephalopathy (10).

Based on the clinical and laboratory findings in the present case, we propose three potentially interacting mechanisms, comprising impaired neurotrophic signaling as a candidate central mechanism and autoimmune and metabolic disturbances as potential modifying factors.

First, impaired neurotrophic signaling may contribute to the development of cognitive dysfunction following IGF-1R inhibition. Downregulated IGF-1 signalling impairs neuronal plasticity and synaptic function in brain regions responsible for memory, attention and executive processes, potentially contributing to manifestations such as disorientation, recent memory impairment, impaired comprehension, and apathy, which closely match the core clinical features of our patient (5, 8, 9). Preclinical studies have linked disruption of IGF-1 signaling to cognitive dysfunction and alterations in cerebral electrophysiological activity, providing a possible biological context for the bilateral diffuse slow-wave activity observed on prolonged EEG monitoring in our patient (11, 12). However, the available evidence does not establish impaired IGF-1 signaling as either a necessary or sufficient mechanism for teprotumumab-associated encephalopathy, and additional immune and metabolic factors may contribute to its clinical expression (10, 13).

Second, disruption of autoimmune homeostasis may represent a potential susceptibility-modifying factor. IGF-1 maintains central immune tolerance by negatively regulating central MHC antigen expression and suppressing excessive activation of autoreactive T cells (14). Pharmacological blockade of IGF-1R may disrupt this immune balance, promoting aberrant exposure of thyroid autoantigens and contributing to immune-mediated neuronal injury (14). Persistently elevated thyroid peroxidase antibodies in our patient most likely reflect the inherent autoimmune background of Graves’ disease rather than *de novo* autoantibody production induced by teprotumumab. Comparison with published cases reveals heterogeneous serological profiles: the patient reported by Hoang (2021) exhibited a transient rise in thyroid autoantibodies after drug exposure with subsequent decline following plasma exchange (5), whereas the case reported by Spicer (2026) had negative thyroid peroxidase and thyroglobulin antibodies (7). Our patient maintained persistently elevated these antibodies throughout the clinical course. These intercase differences indicate marked interindividual heterogeneity in baseline autoimmune background under IGF-1R inhibition. Available pharmacovigilance data are insufficient to confirm that elevated these antibodies represent an independent risk factor for neurological toxicity, the autoimmune background is therefore regarded only as a potential susceptibility-modifying factor rather than a proven pathogenic driver (4, 13, 15).

Third, metabolic disturbance may superimpose and exacerbate neuronal impairment. The IGF-1R exhibits structural and functional interactions with the insulin receptor and can form hybrid receptors (16). IGF-1R blockade disturbs glucose homeostasis and induces insulin resistance, a well-recognised adverse effect of teprotumumab (4, 13). Our patient had completely normal glucose metabolism prior to teprotumumab initiation, with a baseline HbA1c of 5.1 % and fasting plasma glucose of 5.9 mmol/L. Marked new-onset hyperglycaemia emerged after teprotumumab exposure, with an admission HbA1c of 7.7 %, indicating dysglycaemia had persisted for weeks to months. This represented a treatment-secondary metabolic abnormality rather than pre-existing chronic hyperglycaemia, which can impair cerebral energy metabolism and aggravate cognitive and neurobehavioural disturbances (17). Even with complete pre-treatment glycaemic baseline data, this metabolic disturbance is interpreted as a potential aggravating factor rather than the direct primary cause of encephalopathy. Previous literature suggests that advanced age, pre-existing diabetes mellitus and metabolic abnormalities increase the risk of teprotumumab-related hyperglycaemia and other adverse events (16). The case reported by Spicer (2026) had underlying type 2 diabetes mellitus (7), further indicating that metabolic background may modify the risk of neurological adverse events associated with teprotumumab.

Collectively, the available findings support a hypothesis in which teprotumumab-associated encephalopathy may arise from the combined effects of impaired IGF-1R-related neurotrophic signaling, underlying autoimmune susceptibility, and superimposed metabolic disturbance (8, 10, 14, 16). Abnormal neurotrophic signalling may partially explain the patient’s cognitive impairment and electroencephalographic changes, while persistent autoimmune status and drug-induced hyperglycaemia can modify individual susceptibility and disease severity (10, 16, 17). This proposed model remains speculative and should be regarded as a framework for interpreting the present case rather than an established pathogenic sequence.

The first reported patient with teprotumumab-associated encephalopathy developed symptoms after the fourth cycle (5). The patient described by Spicer (2026) experienced symptom onset between the fourth and fifth cycles (7), whereas both our patient and the 62-year-old female case reported by Yee et al. (6) developed central neurotoxic manifestations following the third drug infusion. Williams et al. (18) reviewed published clinical studies and reported that teprotumumab-associated ototoxicity most commonly emerged between the third and sixth infusions. Although ototoxicity and encephalopathy represent distinct neurological adverse events, the temporal overlap suggests that clinically relevant neurological toxicity may become apparent after several treatment cycles rather than exclusively after prolonged exposure. While multiple cases cluster around the third to fifth infusions, the total number of published cases remains very small; therefore, this early infusion cycle cannot yet be defined as a well-established high-risk time window (5–7). Clinicians should maintain heightened vigilance during the early phases of teprotumumab therapy. Prompt systematic evaluation is required whenever patients develop new-onset cognitive, behavioural, or neurological symptoms.

The four globally reported cases share relatively consistent core clinical phenotypes (5–7). All patients exhibited acute-to-subacute onset characterised by rapidly progressive cognitive decline, disorientation, apathy and disturbed sleep architecture, a pattern distinctly different from the chronic, stepwise progression pattern seen in cerebrovascular and neurodegenerative disorders. All patients had preserved neurological function and were independent in activities of daily living before symptom onset, without known pre-existing central nervous system disorders. Unenhanced and contrast-enhanced brain MRI performed in all four patients excluded intracranial haemorrhage, cerebral infarction, space-occupying lesions, and abnormal meningeal or parenchymal enhancement. Brain amyloid angiopathy was identified on MRI in the case reported by Hoang et al. (5), which was interpreted as a pre-existing cerebrovascular change rather than the acute aetiology of the encephalopathic episode. The Spicer et al. (7) case showed minor cerebral T2-weighted hyperintensities consistent with small-vessel ischaemic changes that had no direct association with the acute encephalopathy. No imaging features suggestive of amyloid angiopathy were detected in the Yee et al. (6) case or our patient.

CSF findings demonstrated substantial interindividual variability (5–7). Mild-to-moderate CSF protein elevation was observed in the three prior published cases, whereas CSF opening pressure, cell count and protein level were all within normal reference ranges in our patient. The normal CSF profile in our patient further expands the known CSF phenotypic spectrum of this disorder. The observed heterogeneity in CSF profiles among reported cases can be attributed to three plausible factors. First, individual patients demonstrate variable degrees of blood-brain barrier disruption, which leads to distinct degrees of inflammatory molecule exudation and subsequently contributes to inconsistent CSF biochemical results (19). Second, the time interval from symptom onset to lumbar puncture varies across different cases; dynamic pathological changes in intracranial immune and metabolic status over the disease course may result in inconsistent CSF findings (20). Third, racial and ethnic disparities between Han-Chinese and Caucasian populations may represent an underlying confounding factor (21). Although such ethnic differences cannot be excluded, this hypothesis remains unvalidated owing to the paucity of reported Han-Chinese cases. Comprehensive screening for infectious pathogens, paraneoplastic antibodies and full panels of neuronal autoantibodies yielded negative results in all four patients. This underscores that a systematic exclusion of alternative encephalopathic aetiologies, including infectious, paraneoplastic and other autoimmune encephalitis, is mandatory before attributing encephalopathy to teprotumumab.

Neurological improvement was observed in all four patients after teprotumumab discontinuation and TPE (5–7). During follow-up ranging from 3 weeks to 6 months, most patients achieved sustained cognitive recovery without permanent neurological sequelae, while the therapeutic benefits for thyroid eye disease were maintained. These observations indicate that teprotumumab-associated encephalopathy is potentially reversible with timely recognition and intervention. Nevertheless, it must be emphasised that all available TPE experience in teprotumumab-associated encephalopathy is derived from isolated case reports. TPE cannot be established as a well-recognised rescue or first-line therapy for this condition. Given the limited number of cases and relatively short follow-up duration, definitive conclusions regarding long-term neurological prognosis and the risk of permanent brain injury cannot be drawn.

None of the three previously published cases demonstrated epileptiform discharges on VEEG (5–7). In contrast, prolonged VEEG in our patient showed interictal epileptiform discharges superimposed on a background of bilateral diffuse slow-wave activity, with no clinical convulsions or myoclonus throughout the clinical course. These electrophysiological abnormalities resolved on repeat EEG after short-term symptomatic antiseizure medication therapy. This finding indicates that subclinical cortical hyperexcitability may occur in teprotumumab-associated encephalopathy and expands our neurophysiological understanding of this rare adverse event, analogous to observations in other encephalopathic conditions (22). Our patient had no clinical seizures and did not fulfil diagnostic criteria for non-convulsive status epilepticus. these discharges captured on prolonged VEEG represented subclinical cortical hyperexcitability in the setting of acute encephalopathy rather than ictal electrographic activity. Levetiracetam was therefore initiated neither to treat clinical seizures nor to target ictal discharges. Instead, the goal was to mitigate excessive cortical hyperexcitability during acute encephalopathy with the aim of facilitating cognitive recovery (23). Even in the absence of overt clinical convulsions, interictal epileptiform discharges can disrupt cognitive networks subserving memory and attention and worsen neurological impairment in the context of encephalopathy. By acting on synaptic vesicle protein 2A (SV2A), levetiracetam reduces cortical network hyperexcitability and decreases the burden of interictal epileptiform discharges (23). It may therefore serve as an empirical symptomatic strategy for selected patients with acute encephalopathy and isolated subclinical electrographic abnormalities. This off-label empirical approach lacks high-level evidence and is not equivalent to standard antiseizure treatment for epilepsy.

Williams et al. (18) reported that objective abnormalities associated with IGF-1R inhibition may occur in the absence of corresponding subjective symptoms. Specifically, audiological abnormalities were identified in some patients without overt hearing complaints. By analogy, one may cautiously hypothesise that teprotumumab-associated encephalopathy can present with isolated subclinical cortical hyperexcitability on VEEG without clinical convulsions (18). Nevertheless, this remains a tentative clinical analogy, and the definitive pathophysiological link between these two phenomena has not been established. This suggests that prolonged VEEG may be considered selectively for patients developing unexplained new-onset cognitive-behavioural disturbances during teprotumumab therapy, particularly when subclinical cortical electrical abnormalities are suspected.

Notably, for this category of drug-induced encephalopathy, antiseizure agents exert merely symptomatic benefits, and definitive disease resolution depends on interventions directed against the inciting primary pathological insult (24). Insulin, levetiracetam and TPE were initiated nearly concurrently in this patient. The substantial temporal overlap of multiple therapies prevents separation of the individual contribution of each intervention to neurological recovery based on clinical chronology alone. Accordingly, this case cannot demonstrate that therapeutic plasma exchange is superior to levetiracetam, this limitation should be acknowledged objectively.

Our patient had completely normal baseline glucose metabolism before teprotumumab administration, with a baseline HbA1c of 5.1% and a fasting plasma glucose level of 5.9 mmol/L, confirming hyperglycaemia as a new-onset adverse event secondary to teprotumumab exposure. Despite the development of treatment-emergent hyperglycaemia, cumulative clinical and laboratory evidence excludes isolated hyperglycaemia or hyperosmolar-hyperglycaemic syndrome as the primary cause of the patient’s encephalopathy. Biochemical assessment on admission yielded a calculated plasma osmolality of 294.87 mOsm/kg, which was below the conventional diagnostic threshold for hyperosmolar-hyperglycaemic syndrome (> 320 mOsm/kg), and urine ketone testing was negative, collectively ruling out the typical laboratory profile of hyperosmolar-ketotic metabolic encephalopathy (25, 26). Temporal clinical observations further contradicted a primary metabolic aetiology: immediate insulin administration at hospital admission effectively lowered blood glucose levels within one day, whereas the patient’s cognitive and behavioural encephalopathic symptoms remained refractory to glycaemic correction. Substantial resolution of neuropsychiatric abnormalities was only achieved following the first session of therapeutic plasma exchange (25). A purely hyperglycaemia-driven encephalopathy would be expected to improve synchronously with normalized glucose levels, a pattern inconsistent with the clinical course observed herein. Furthermore, pure metabolic hyperosmolar-hyperglycaemic encephalopathy is insufficient to account for the full spectrum of the patient’s clinical manifestations, which included prominent apathy, bradyphrenia, reduced spontaneous speech, and electrographic abnormalities characterised by diffuse slow-wave activity combined with interictal epileptiform discharges; such multifaceted neurological and electrophysiological alterations are not typical of uncomplicated metabolic encephalopathy, which usually resolves concurrently with metabolic recovery (25). Nevertheless, the nearly simultaneous initiation of insulin therapy and therapeutic plasma exchange created overlapping therapeutic time windows, making it difficult to quantitatively isolate the independent contribution of glycaemic improvement to neurological recovery. The previously reported case from Spicer et al. (7) with type 2 diabetes mellitus further supports that hyperglycaemia functions as an additive aggravating factor rather than the exclusive pathogenic driver of teprotumumab-associated encephalopathy.

A comprehensive and systematic diagnostic workup was conducted in the present case to rule out alternative aetiologies of rapidly progressive cognitive and behavioural deterioration, Hashimoto’s encephalopathy was prioritised as a critical differential diagnosis given the persistently elevated thyroid autoantibodies detected in the patient, alongside prior phase III clinical trial observations of suspected teprotumumab-associated immune-mediated neurological events linked to thyroid autoimmunity (1, 15, 27). Characterised by heterogeneous multi-domain cognitive and behavioural dysfunction, Hashimoto’s encephalopathy may present with entirely normal brain imaging, and seizures or myoclonus are not mandatory diagnostic features. In this case, the absence of myoclonus and clinical seizures, unremarkable white matter changes on brain MRI, and rapid neurological recovery following teprotumumab discontinuation combined with therapeutic plasma exchange without glucocorticoid intervention collectively make Hashimoto’s encephalopathy a less probable diagnosis, aligning with the diagnostic logic applied in previously reported teprotumumab-associated encephalopathy cases (5, 6, 27). Notably, clinical improvement after plasma exchange cannot reliably distinguish teprotumumab-induced encephalopathy from other immune-mediated encephalopathies, as therapeutic response to this intervention is non-specific, and definitive aetiological attribution depends on thorough exclusion of alternative disorders combined with the distinct temporal correlation between drug exposure and symptom onset. Distinct thyroid autoantibody dynamic patterns have been observed across available cases, strongly suggesting interindividual heterogeneity in immune profiles under IGF-1R inhibition. The first reported case by Hoang et al. (5) exhibited transient post-treatment thyroid autoantibody elevation that gradually declined following plasma exchange, which was speculated to represent temporary modulation of autoreactive immune responses during systemic IGF-1R blockade. In contrast, our patient demonstrated persistently elevated thyroid autoantibody levels throughout the disease course, without a treatment-induced serological surge, a pattern more likely attributable to the pre-existing autoimmune background of Graves’ disease and thyroid eye disease rather than *de novo* serological alteration triggered by teprotumumab (14, 15). Despite these observable serological variations, current evidence remains insufficient to validate a direct causal relationship between thyroid autoantibody titres and the pathogenesis of teprotumumab-associated encephalopathy. Although baseline thyroid autoantibody measurement and longitudinal serological follow-up may be implemented in future case series to clarify potential correlations, such stratification remains a research-focused strategy rather than a conventional clinical practice.

Further differential diagnostic considerations included autoimmune catatonia and non-convulsive status epilepticus, both of which may manifest with acute behavioural and cognitive disturbances. Although catatonic syndromes secondary to autoimmune encephalitis can present with apathy, mutism, and psychomotor retardation that partially overlap with the patient’s clinical profile, classic core catatonic features, such as stereotyped posturing and behavioural negativism, were entirely absent. Combined with negative results from extensive immune and infectious workups, primary catatonia was therefore unlikely (28). Similarly, prolonged video-electroencephalography effectively ruled out non-convulsive status epilepticus; the recorded electrographic changes were limited to isolated interictal epileptiform discharges without sustained rhythmic ictal activity, which did not meet the established diagnostic criteria for non-convulsive status epilepticus (29).

Systematic laboratory and auxiliary examinations further excluded toxic-metabolic encephalopathy. Comprehensive assessments of plasma osmolality, ketone bodies, serum ammonia, electrolytes, vitamin profiles and infectious indicators eliminated common metabolic and infectious triggers of acute encephalopathy. Consistent with the aforementioned glycometabolic analysis, the definitively normal pretreatment glucose baseline confirmed that hyperglycaemia was a secondary adverse event induced by teprotumumab treatment, acting merely as a superimposed aggravating factor rather than the primary pathogenic cause of acute encephalopathy in this patient. Based on the clear temporal association between teprotumumab exposure and acute encephalopathy onset, the rigorous exclusion of alternative infectious, autoimmune and metabolic aetiologies, and the prominent clinical recovery after targeted plasma exchange therapy, a standardized causality evaluation was performed using the Naranjo Adverse Drug Reaction Probability Scale (30). The total score of 6 obtained in this assessment corresponds to a probable adverse drug reaction grade (**Supplementary Table 4**). Collectively, the multi-dimensional clinical, laboratory and therapeutic evidence strongly supports the final diagnosis of teprotumumab-induced encephalopathy in the present case.

The three previously reported cases all adopted an every-other-day therapeutic plasma exchange (TPE) regimen with a planned total of five sessions (5–7). The patient in Hoang’s report switched to TPE after failing to respond to prior glucocorticoid and intravenous immunoglobulin (IVIG) therapy. The patient described by Yee et al. (6) experienced transient neurological symptom fluctuation after the first TPE session, suggesting that circulating putative pathogenic mediators may rebound during treatment-free intervals under the alternate-day protocol, carrying a risk of clinical relapse. The patient described by Spicer et al. (7) received olanzapine in addition to TPE for symptomatic management of psychiatric manifestations.

Drawing on clinical experience from these published cases, an individualised once-daily TPE regimen was selected for our patient for the following reasons. First, consistent with the observation from the case reported by Yee et al. (6), transient symptom fluctuation may occur following alternate-day TPE. We hypothesised that the concentrations of putative circulating pathogenic agents could rebound during inter-treatment intervals and trigger clinical relapse. To achieve more continuous and stable clearance of circulating harmful mediators and minimise the time window for mediator rebound, the alternate-day schedule was abandoned in favour of daily TPE. Second, our patient presented with rapidly progressive neurocognitive impairment involving extensive multi-domain cognitive and behavioural dysfunction, indicating severe acute encephalopathy. An intensified treatment frequency was chosen with the aim of achieving more rapid control of the acute encephalopathic state. Third, based on the experience from the index published case showing lack of efficacy of high-dose glucocorticoids, high-dose steroid pulse therapy was omitted, and TPE was adopted as the primary immunomodulatory intervention (5).

Our patient developed an acute infusion-related anaphylactoid reaction immediately upon initiation of the third TPE session, forcing premature termination of treatment. Nevertheless, marked cognitive improvement was achieved after only two complete TPE sessions. Direct evidence confirming that circulating pathogenic autoantibodies or immune complexes mediate teprotumumab-associated encephalopathy is currently absent. It should be emphasised that given the very limited number of reported cases, TPE cannot be regarded as an established first-line disease-specific therapy, and no consensus exists regarding the optimal number of sessions or treatment intervals. Our experience suggests that the TPE regimen should be individualised according to clinical response and patient tolerance rather than rigid adherence to a fixed five-session alternate-day protocol. Clinicians must remain vigilant for infusion-related hypersensitivity and other adverse events during TPE, with close monitoring throughout the procedure and prepared protocols for complication management (31).

Individualized symptomatic and supportive management is essential for patients with teprotumumab-associated encephalopathy. For patients presenting with EEG-detected interictal epileptiform discharges, short-term antiseizure medication can be administered tailored to clinical manifestations and electrographic features. Clinically significant teprotumumab-related hyperglycaemia requires timely recognition and prompt intervention with individualized glucose-lowering strategies, including insulin therapy when necessary, to mitigate subsequent metabolic damage and complications. Additionally, targeted supportive care is recommended for neuropsychiatric symptoms such as apathy and insomnia, with symptomatic medications gradually tapered or discontinued dynamically based on the patient’s ongoing neurological recovery and overall clinical progress.

The strengths of this report include the first documentation of teprotumumab-associated encephalopathy in a Han-Chinese patient, comprehensive exclusion of alternative aetiologies, structured causality assessment using Naranjo, and a detailed CARE-compliant timeline. Several limitations of the present study should be acknowledged. First, this single-case analysis, with only three comparable published cases for reference, is constrained by an extremely small sample size, which precludes robust cross-case comparisons and definitive conclusions regarding the causal relationships among immune dysregulation, metabolic abnormalities, and clinical phenotypes. The interindividual heterogeneity in thyroid autoantibody and CSF profiles observed across cases remains speculative with limited generalizability. Furthermore, although the Naranjo scale verified a probable adverse drug reaction, this tool only provides probabilistic assessment and cannot establish causality between teprotumumab and encephalopathy. Second, all mechanistic interpretations are indirectly deduced from clinical manifestations, neuroimaging, VEEG recordings, and routine laboratory data, without direct detection of cerebral IGF-1R signaling, intracranial inflammatory activity, or cerebral metabolic status. As such, the proposed pathogenic model involving IGF-1R dysfunction, immune perturbation, and metabolic aggravation requires further validation. Third, concurrent administration of insulin, levetiracetam, and therapeutic plasma exchange made it impossible to distinguish the independent therapeutic contribution of each intervention to neurological recovery. The relative efficacy of plasma exchange and the specific neuroprotective effect of glycemic correction could not be precisely determined, and inconsistent treatment regimens in prior cases further hindered accurate therapeutic attribution. Fourth, baseline standardized neuropsychological assessment before teprotumumab initiation was not performed. Only MMSE was applied during hospitalization, which has low sensitivity for subtle executive dysfunction; incomplete cognitive testing restricted comprehensive phenotypic evaluation of cognitive impairment. Fifth, free teprotumumab and pathogenic autoantibody levels in plasma exchange effluent were not measured, leading to a lack of direct molecular evidence verifying the clearance mechanism of plasma exchange. Sixth, the absence of HLA genotyping prevented exploration of genetic autoimmune susceptibility underlying the unique vulnerability of Han-Chinese patients to this complication. Seventh, post-treatment orbital MRI was not performed, and facial photographs were not obtained because the patient did not provide consent for facial photography. Hertel exophthalmometry measurements and formal soft-tissue inflammation assessment were not systematically recorded. Therefore, documentation of TED improvement is limited to the patient’s subjective clinical report and routine clinical observation. Finally, due to the limited number of reported cases and short follow-up duration, unified diagnostic criteria, optimal treatment strategies, long-term prognostic profiles, and standardized risk stratification protocols for teprotumumab-associated encephalopathy have not been established. Further multicenter case accumulation, prospective longitudinal follow-up, and in-depth mechanistic studies are needed to fill these research gaps.

## 4 Conclusion

This study reports the fourth global case and the first Han-Chinese case of teprotumumab-associated encephalopathy, further expanding the clinical and serological phenotypic spectrum of this rare neurological adverse event. Through comprehensive clinical evaluation and rigorous differential diagnosis, this work systematically excludes multiple common causes of acute encephalopathy, including infectious, toxic-metabolic, and other autoimmune etiologies, and confirms that teprotumumab-related hyperglycemia is a treatment-emergent secondary change that only serves as an aggravating factor rather than the primary cause of encephalopathy. Distinct from the transient thyroid autoantibody fluctuations observed in previously reported Caucasian cases, sustained elevation of thyroid autoantibodies was identified in this Han-Chinese patient, further demonstrating obvious ethnic heterogeneity in serological characteristics following IGF-1R blockade. The patient’s neurological symptoms improved markedly after discontinuation of teprotumumab combined with comprehensive symptomatic and supportive treatment including plasma exchange, indicating the clinical value of timely intervention and multidisciplinary management for this rare adverse event. Given the currently limited case volume and insufficient recognition of teprotumumab-associated encephalopathy, standardized neurological monitoring during medication is essential to facilitate early identification and timely intervention of atypical neuropsychiatric symptoms. Future multicenter prospective studies with larger sample sizes are required to clarify the underlying pathogenesis, establish unified diagnostic criteria, and optimize standardized management and risk stratification strategies for this rare drug-induced neurological complication.

## Data Availability

The original contributions presented in the study are included in the article/**Supplementary Material**. Further inquiries can be directed to the corresponding author.
