# Supplementary material for: Rapidly progressive cognitive decline and drug-induced encephalopathy secondary to teprotumumab for thyroid eye disease: a case report and narrative literature review
Source: Front Immunol. 2026 Oct 22;17:1985279. doi: 10.3389/fimmu.2026.1985279 (PMC13736744; doi:10.3389/fimmu.2026.1985279)
Supplement: Supplementary file 1 [file DataSheet1.pdf]

Supplementary Table 3. Timeline of clinical manifestations, interventions, functional recovery, and laboratory findings in the present case of teprotumumab-associated encephalopathy

Time point	Key clinical events	Interventions	Cognitive and functional status	Key imaging, EEG, and laboratory findings	Glycemic and metabolic findings
Before encephalopathy onset (teprotumumab cycles 1–3)	The patient had thyroid eye disease (TED) with photophobia, lacrimation, strabismus, diplopia, and marked bilateral proptosis. No behavioral or cognitive abnormalities were observed, and he remained fully independent.	Teprotumumab was administered intravenously for TED. Long-term therapy included methimazole 5 mg every other day and fenofibrate 0.1 g once daily.	No standardized cognitive assessment was performed. Clinical evaluation indicated intact cognition and independence in all basic and instrumental activities of daily living (ADL).	Orbital MRI showed extraocular muscle enlargement consistent with TED. Brain MRI was normal, and EEG was not performed. During treatment, high-sensitivity thyroid-stimulating hormone (hs-TSH) and triglyceride levels decreased; thyroid autoantibodies remained elevated; free triiodothyronine (FT3) and free thyroxine (FT4) remained within reference ranges.	HbA1c, 5.1%; fasting plasma glucose (FPG), 5.9 mmol/L.
20 days after the third infusion (admission)	Acute, rapidly progressive behavioral and neurocognitive dysfunction developed on awakening, including apathy, bradyphrenia, markedly reduced spontaneous speech, generalized rigidity, impaired verbal comprehension, recent-memory impairment, refusal of food and medication, and insomnia. Daily functioning declined markedly.	Teprotumumab was permanently discontinued. Methimazole and fenofibrate were continued.	MMSE score was 15/30, with deficits in orientation, repetition, reading, writing, and visuoconstruction. Walking and toileting remained independent, but feeding and dressing required assistance; all instrumental ADL were impaired. On AVLT learning trials, the patient recalled 0, 0, and 2 words; delayed recall and recognition were not assessed.	Emergency brain MRI excluded hemorrhage, infarction, and mass lesions. Infectious, autoimmune, neoplastic, and nutritional screening was unrevealing. Sodium was 138.31 mmol/L, urea 4.12 mmol/L, and calculated plasma osmolality 294.87 mOsm/kg (reference range, 275–295), not supporting hyperosmolar encephalopathy.	New-onset hyperglycemia: HbA1c, 7.7%; FPG, 14.13 mmol/L.

Time point	Key clinical events	Interventions	Cognitive and functional status	Key imaging, EEG, and laboratory findings	Glycemic and metabolic findings
Hospital day 1	Hyperglycemia remained poorly controlled. Mild glycemic improvement after symptomatic treatment was not accompanied by improvement in the encephalopathy.	Basal-bolus insulin was initiated. Levetiracetam 0.5 g twice daily was started for subclinical cortical hyperexcitability. Methimazole and fenofibrate were continued.	Cognitive and behavioral status remained unchanged from admission, with persistent multidomain impairment.	Contrast-enhanced brain MRI showed no abnormal meningeal or parenchymal enhancement. Video EEG showed a poorly regulated 8–9-Hz posterior dominant rhythm, diffuse theta-delta slowing, and bilateral interictal epileptiform discharges without electrographic or clinical seizures. CSF and serum autoimmune encephalitis panels were negative, and CSF metagenomic next-generation sequencing (mNGS) detected no pathogen.	FPG, 14.2 mmol/L; 2-h postprandial glucose, 10.7–19.1 mmol/L.
Hospital day 2	Blood glucose decreased slightly. Marked improvement in the encephalopathic syndrome occurred after the first therapeutic plasma exchange (TPE).	The first once-daily TPE session was completed.	Rigidity resolved, sleep normalized, spontaneous speech increased, and independent feeding and dressing returned. Responses to simple commands were intact, although mild temporal and spatial disorientation persisted.	No new abnormalities were identified on imaging or laboratory testing.	FPG, 10.3 mmol/L; 2-h postprandial glucose, 10.0–12.4 mmol/L.
Hospital day 3	Neurological improvement continued, with further recovery of orientation. TPE was well tolerated.	The second once-daily TPE session was completed without complications.	Temporal and spatial orientation improved substantially, with continued recovery of overall cognitive function.	Repeat brain MRI showed no new intracranial lesions.	FPG, 8.4 mmol/L; 2-h postprandial glucose, 10.6–14.9 mmol/L.

Time point	Key clinical events	Interventions	Cognitive and functional status	Key imaging, EEG, and laboratory findings	Glycemic and metabolic findings
Hospital day 4	The third TPE session was interrupted by an acute infusion-related reaction with fever, chills, and hypotension, without urticaria, angioedema, or respiratory compromise. Cognitive improvement had already stabilized after the first two sessions.	Intravenous fluids and low-dose corticosteroids were administered. The third TPE session was terminated, and further sessions were discontinued.	Cognitive and behavioral function continued to improve without fluctuation despite discontinuation of TPE.	No new abnormalities were identified.	FPG, 10.7 mmol/L; 2-h postprandial glucose, 9.3–12.2 mmol/L.
Hospital day 5	Encephalopathy-related symptoms continued to improve, and the clinical condition remained stable.	Insulin, levetiracetam, and baseline medications were continued.	Cognitive and behavioral recovery remained stable without deterioration or recurrence.	No additional imaging or EEG was performed.	FPG, 8.8 mmol/L; 2-h postprandial glucose, 6.4–9.9 mmol/L.
Hospital day 8	Neurological function improved markedly, with substantial resolution of encephalopathic manifestations.	Insulin and levetiracetam were continued.	MMSE score improved to 25/30. Orientation, memory, and verbal function nearly normalized; mild deficits remained in serial subtraction and multistep executive tasks. Basic ADL fully recovered, while some instrumental ADL remained impaired.	Repeat video EEG showed normalization of background activity and resolution of diffuse slowing and interictal epileptiform discharges. Predischarge brain MRI showed no new structural or ischemic lesions.	FPG, 8.4 mmol/L; 2-h postprandial glucose, 8.7–11.7 mmol/L.
Hospital day 9 (discharge)	Neurological recovery was favorable, and the patient met discharge criteria with stable vital signs and mental status.	Levetiracetam was discontinued. Outpatient treatment for glycemic control and thyroid disease was continued.	Cognitive and behavioral improvement was sustained.	No additional imaging, EEG, or laboratory examinations were performed before discharge.	Blood glucose was not measured.

Time point	Key clinical events	Interventions	Cognitive and functional status	Key imaging, EEG, and laboratory findings	Glycemic and metabolic findings
2-month outpatient follow-up	The improvement in TED remained stable, with reduced proptosis and diplopia. Encephalopathy did not recur, and no additional immunomodulatory treatment was administered.	Endocrinology follow-up and baseline medications were continued. No further TPE or other immunological intervention was performed.	Cognitive function recovered completely without residual deficits. Independence in all basic and instrumental ADL was restored.	Follow-up EEG was normal. hs-TSH decreased further to 5.715 mIU/mL; FT3 and FT4 remained within reference ranges; thyroid autoantibodies remained elevated.	HbA1c, 5.8%; FPG, 6.89 mmol/L.

Abbreviations: ADL, activities of daily living; AVLT, Auditory Verbal Learning Test; CSF, cerebrospinal fluid; EEG, electroencephalography; FPG, fasting plasma glucose; FT3, free triiodothyronine; FT4, free thyroxine; HbA1c, glycated hemoglobin; hs-TSH, high-sensitivity thyroid-stimulating hormone; MMSE, Mini-Mental State Examination; mNGS, metagenomic next-generation sequencing; MRI, magnetic resonance imaging; TED, thyroid eye disease; TPE, therapeutic plasma exchange. **Note:** The timeline extends from before encephalopathy onset through the 2-month outpatient follow-up
