# Supplementary material for: Rapidly progressive cognitive decline and drug-induced encephalopathy secondary to teprotumumab for thyroid eye disease: a case report and narrative literature review
Source: Front Immunol. 2026 Oct 22;17:1985279. doi: 10.3389/fimmu.2026.1985279 (PMC13736744; doi:10.3389/fimmu.2026.1985279)
Supplement: Supplementary file 2 [file DataSheet2.pdf]

Supplementary Table 4. Naranjo Adverse Drug Reaction Probability Scale for the present case of teprotumumab-associated encephalopathy

No.	Question	Yes	No	Unknown	Case score
1	Are there previous conclusive reports of this reaction?	+1	0	0	+1
2	Did the adverse event occur after the suspected drug was administered?	+2	−1	0	+2
3	Did the adverse reaction improve when the drug was discontinued or a specific antagonist was administered?	+1	0	0	+1
4	Did the adverse reaction recur when the drug was readministered?	+2	−1	0	0
5	Are there alternative causes, other than the drug, that could independently have caused the reaction?	−1	+2	0	+2
6	Did the reaction recur when a placebo was administered?	−1	+1	0	0
7	Was the drug detected in blood or other body fluids at concentrations known to be toxic?	+1	0	0	0
8	Was the reaction more severe when the dose was increased or less severe when the dose was decreased?	+1	0	0	0
9	Did the patient have a similar reaction to the same or a related drug during previous exposure?	+1	0	0	0
10	Was the adverse event confirmed by objective evidence?	+1	0	0	+1
	**Total score**				**6**

Score interpretation: ≥9, definite adverse drug reaction; 5–8, probable; 1–4, possible; ≤0, doubtful. The total score of 6 indicates a probable adverse drug reaction. A score of 0 indicates that the criterion was not applicable or could not be assessed in this case. **Source:** Adapted from Naranjo CA, Busto U, Sellers EM, et al. A method for estimating the probability of adverse drug reactions. Clin Pharmacol Ther. 1981;30(2):239–245.
