# Supplementary material for: Rapidly progressive cognitive decline and drug-induced encephalopathy secondary to teprotumumab for thyroid eye disease: a case report and narrative literature review
Source: Front Immunol. 2026 Oct 22;17:1985279. doi: 10.3389/fimmu.2026.1985279 (PMC13736744; doi:10.3389/fimmu.2026.1985279)
Supplement: Supplementary file 3 [file DataSheet3.pdf]

CARE Checklist

Manuscript title:

Rapidly Progressive Cognitive Decline and Drug-Induced Encephalopathy Secondary to Teprotumumab for Thyroid Eye Disease: A Case Report and **Narrative Literature Review**

Target journal: Frontiers in Immunology

Article type: Case Report

**CARE item**	**Checklist requirement**	**Status**	**Location in current manuscript / action required**
**1. Title**	The diagnosis or intervention of primary focus followed by “case report”	**Reported**	**Title.** The title clearly identifies the manuscript as a case report.
**2. Keywords**	2–5 keywords identifying diagnoses/interventions, including “case report”	**reported**	Keywords are provided in the Abstract. Keywords include teprotumumab, thyroid eye disease, drug-induced encephalopathy, IGF-1R and therapeutic plasma exchange.
**3a. Abstract – Introduction**	What is unique about the case and what it adds to the literature	**Reported**	**Abstract, Introduction.** The rarity of teprotumumab-associated encephalopathy and the limited number of previous cases are described.
**3b. Abstract – Main**	Main symptoms and/or important clinical	**Reported**	**Abstract, Case Presentation.** Acute apathy, cognitive

**symptoms/findings**	findings		impairment, MMSE score, EEG findings, hyperglycemia, and thyroid autoantibody elevation are summarized.
**3c. Abstract – Diagnosis/interventions/outcomes**	Main diagnosis, therapeutic interventions, and outcomes	**Reported**	**Abstract, Case Presentation.** Teprotumumab discontinuation, TPE, neurological improvement, EEG normalization, and 2-month outcome are reported.
**3d. Abstract – Conclusion**	Main take-away lesson from the case	**Reported**	**Abstract, Conclusions.** Early recognition of neuropsychiatric symptoms during teprotumumab therapy, prompt drug discontinuation, and individualised management are the main take-away lessons.
**4. Introduction**	Brief background explaining why the case is unique	**Reported**	**Introduction.** The rarity and potential severity of teprotumumab-associated encephalopathy are described.
**5a. Patient information**	De-identified demographic and other patient-specific information	**Reported**	**Case Presentation.** A 56-year-old Chinese man with TED is described.
**5b. Main concerns and symptoms**	Patient's main concerns and symptoms	**Reported**	**Case Presentation.** Apathy, bradyphrenia, reduced speech, memory impairment, impaired comprehension, rigidity, refusal of food/medication, and insomnia are described.
**5c. Medical, family, and psychosocial history**	Relevant medical, family, and psychosocial history, including genetic information	**reported**	**Case Presentation.** Relevant medical history, including Graves' disease, hyperlipidemia, absence of

	when appropriate		diabetes and neurodegenerative disease, and baseline independence, is reported. Smoking history (non-smoker) is now reported. Family and psychosocial history were not clinically relevant to this case and are therefore not reported.
**5d. Relevant past interventions**	Relevant previous interventions and their outcomes	**Reported**	**Case Presentation.** Long-term methimazole and fenofibrate treatment and initiation of teprotumumab are described.
**6. Clinical findings**	Relevant physical examination and other significant clinical findings	**Reported**	**Case Presentation.** Neurological examination, orientation, affect, speech, and MMSE score are reported.
**7. Timeline**	Important historical and current information organized as a timeline	**Partially reported**	**Case Presentation.** Dates and sequence of events are described narratively. Supplementary Table 3. A CARE-compliant timeline of clinical manifestations, interventions, functional recovery, and laboratory findings has been added.
**8a. Diagnostic assessment – Methods**	Diagnostic methods, including examination, laboratory testing, imaging, surveys, etc.	**Reported**	**Case Presentation.** MRI, contrast-enhanced MRI, continuous EEG, CSF examination, infectious testing, autoimmune encephalitis antibodies, toxicology, metabolic testing, rheumatological testing, and thyroid-related tests are

			reported.
**8b. Diagnostic challenges**	Diagnostic challenges, including access, financial, or cultural issues when relevant	**Partially reported**	**Case Presentation.** Diagnostic uncertainty and differential diagnosis are discussed, particularly Hashimoto's encephalopathy. Diagnostic challenges are now explicitly discussed, including the limitations of negative neuronal-surface antibody testing, normal MRI, and nonspecific thyroid antibody elevation in excluding alternative diagnoses.
**8c. Diagnosis and differential diagnosis**	Diagnosis, including other diagnoses considered	**Reported**	**Case Presentation and Discussion.** Teprotumumab-associated immune-mediated encephalopathy is considered the most likely diagnosis; Hashimoto's encephalopathy and infectious, autoimmune, metabolic, and structural causes are discussed.
**8d. Prognostic characteristics**	Prognostic characteristics when applicable	**Not applicable / limited**	No validated prognostic characteristics exist for this extremely rare adverse event. Clinical outcome is reported in the follow-up section.
**9a. Therapeutic intervention – Types**	Types of intervention, including pharmacological, surgical, preventive, or self-care	**Reported**	**Case Presentation.** Teprotumumab discontinuation, TPE, insulin, levetiracetam, methimazole, fenofibrate, intravenous fluids, and supportive treatment are described.

**9b. Therapeutic intervention – Administration**	Dosage, strength, duration, and frequency	**Reported**	**Case Presentation.** Drug doses and frequencies are reported. TPE was planned once daily for three consecutive days.
**9c. Changes in intervention**	Changes in intervention and rationale	**Reported**	**Case Presentation.** Teprotumumab was permanently discontinued. The third TPE session was terminated because of acute hypersensitivity. Levetiracetam was subsequently withdrawn after recovery.
**10a. Follow-up and outcomes**	Clinician- and patient-assessed outcomes	**Reported**	**Case Presentation.** MMSE improved from 15 to 25, neurological symptoms improved, and no residual neurological sequelae were reported at 2 months.
**10b. Follow-up tests**	Important follow-up diagnostic and other test results	**Reported**	**Case Presentation.** Repeat EEG, brain MRI, thyroid function tests, thyroid autoantibodies, and clinical follow-up are reported.
**10c. Intervention adherence and tolerability**	Adherence and tolerability and how they were assessed	**Partially reported**	**Case Presentation and Discussion.** TPE intolerance due to hypersensitivity reaction is described. Overall treatment adherence and tolerability are now explicitly discussed.
**10d. Adverse and unanticipated events**	Adverse or unanticipated events	**Reported**	**Case Presentation.** Acute hypersensitivity during TPE and clinically significant hyperglycemia during

			teprotumumab treatment are reported.
**11a. Discussion – Strengths and limitations**	Strengths and limitations of the case	**reported**	**Discussion.** Limitations are described extensively. Strengths (first Han-Chinese case, comprehensive differential diagnosis, structured causality assessment, detailed timeline) and limitations (single case, no formal cognitive baseline, no post-treatment orbital MRI, overlapping therapeutic windows) are now both explicitly summarised.
**11b. Discussion – Relevant literature**	Relevant medical literature	**Reported**	**Discussion.**The case is compared with the three previously reported cases and relevant pharmacological, immunological, metabolic, and safety literature.
**11c. Discussion – Scientific rationale**	Scientific rationale for conclusions, including possible causes	**Reported**	**Discussion.** Potential contributions of altered IGF-1R-related neurotrophic signaling, autoimmune susceptibility, and metabolic disturbance are discussed with appropriate caution.
**11d. Discussion – Take-away lessons**	Main take-away lessons	**Reported**	**Discussion.** Recognition of new neuropsychiatric symptoms, appropriate diagnostic evaluation, glucose monitoring, EEG assessment when indicated, and individualised management.

**12. Patient perspective**	Patient perspective or experience whenever possible	**Not applicable**	No Patient Perspective section is currently included.
**13. Informed consent**	Patient gave informed consent for publication	**Reported**	**Ethics statement.** Written informed consent for publication has been obtained from the patient. Add the following statement to the manuscript: "Written informed consent was obtained from the patient for the publication of this case report and any potentially identifiable clinical data and images included in this article."
